# Exploratory integrative analysis of infant gut microbiome and metabolome suggests diet- and microbiome-derived metabolites associated with childhood asthma

**DOI:** 10.1007/s11306-026-02540-3

**Published:** 2026-09-27

**Authors:** Kedir N. Turi, Xiliu Wu, Yajing Li, Christian Rosas-Salazar, Tebeb Gebretsadik, Meghan H. Shilts, Suman R. Das, James Gern, Yaomin Xu, Tina V. Hartert

**Affiliations:** 1 Department of Epidemiology and Biostatistics, Indiana University, Bloomington, IN USA; 2 Department of Health Data Science & Biostatistics, Peter O’Donnell Jr. School of Public Health, UT Southwestern Medical Center, Dallas, USA; 3 Department of Pediatrics, Vanderbilt University Medical Center, Nashville, TN USA; 4 Department of Biostatistics, Vanderbilt University Medical Center, Nashville, TN USA; 5 Department of Medicine, Vanderbilt University Medical Center, Nashville, TN USA; 6 Department of Pediatrics, University of Wisconsin, Madison, WI 53705 USA; 7 Epidemiology and Biostatistics, School of Public Health, Indiana University Bloomington, 1025 E 7th St PH 029B, Bloomington, IN 47405 USA

**Keywords:** Asthma, Multi-omics integration, Gut microbiome, Metabolomics, Microbial metabolism, Diet-derived pathways

## Abstract

**Background:**

Early-life gut microbiome composition has been linked to childhood asthma risk, but the metabolic pathways underlying this association remain unclear.

**Objective:**

To integrate infant gut microbiome and metabolome data to identify pathways associated with asthma risk.

**Methods:**

Participants were from the INSPIRE birth cohort with early infancy 16S rRNA microbiome data (n=402), urinary metabolomics (n=199), and stool metabolomics (n=58). Overlapping datasets included 89 (urine) and 58 (stool) infants. Current asthma at age 6 was assessed by validated questionnaires. Nested sparse partial least squares discriminant analysis prioritized metabolites and amplicon sequence variants (ASVs) feature selection, followed by Spearman rank correlations to evaluate metabolite-microbiome associations with Bonferroni correction.

**Results:**

Several significant correlations were identified in associations between the stool microbiome and metabolites, primarily involving lipid-related metabolites and microbiome taxa such as *Staphylococcus* sp. (unclassified)*, Enterobacteriaceae* sp. (unclassified)*, Veillonella* sp. (unclassified)*, Thomasclavelia ramosa, Bacteroides* sp.(unclassified), and *Clostridium innocuum* group. The correlations between stool microbiome and urine metabolites were weak. Many associated metabolites are likely diet-derived, with microbiome co-metabolising.

**Conclusion:**

Exploratory microbiome-metabolite correlations suggest diet-related metabolites may link the infant gut environment to later asthma risk.

**Supplementary Information:**

The online version contains supplementary material available at https://doi.org/10.1007/s11306-026-02540-3.

## Highlights

Exploratory correlations between prioritized infant gut taxa and metabolites generate hypotheses for future studies of early-life asthma development.

**Supplementary Information:**

The online version contains supplementary material available at https://doi.org/10.1007/s11306-026-02540-3.

## Introduction

Development of childhood asthma is a complex and multifactorial process that begins in utero involving interactions between genetic and environmental factors (Vercelli 2004; Hernandez-Pacheco et al. 2022). Early life gut microbiota plays an important role in the pathogenesis of childhood asthma (Fujimura and Lynch 2015; Jatzlauk et al. 2017). A landmark study from the Canadian CHILD cohort examined stool samples from 319 infants and found that children at high risk for asthma exhibited transient gut microbial dysbiosis during the first 100 days of life (Arrieta et al. 2015). Specifically, reduced abundance of four bacterial genera, *Faecalibacterium, Lachnospira, Veillonella, and Rothia* (collectively termed FLVR), was associated with increased risk of wheeze and atopy, early indicators of childhood asthma. Subsequent studies have replicated these findings. In the Copenhagen Prospective Studies on Asthma in Childhood cohort, delayed maturation of the gut microbiome at one year of age was associated with increased asthma risk by age five. Children with asthma exhibited evidence of microbial immaturity and lower abundance of several beneficial taxa involved in immune regulation (Stokholm et al. 2018). However, our understanding of how the gut microbiota is involved in respiratory diseases such as asthma is limited.

Bacteria serve several functions in the gut, including fermentation of food, protection against pathogens, stimulating immune responses, and vitamin production (Macia et al. 2015; Hillman et al. 2017; Takeuchi and Ohno 2021). Direct and indirect interaction between gut microbiota and the local immune system, including gut microbiota’s role in maintaining the balance of pro-inflammatory and regulatory signals, has been the focus of several studies (Skelly et al. 2019; Karmarkar and Rock 2013; Balmer et al. 2014; Fulde et al. 2018). The recently proposed concept of the gut-lung axis highlights the important communication between the gut microbiome and pulmonary health, emphasizing the systemic impact of gut microbial communities and their far-reaching effects (Enaud et al. 2020). Evidence highlighting a greater prevalence of respiratory diseases in patients with inflammatory gut conditions (Ji et al. 2014) further supports a connection between the gut and respiratory systems. A hypothesised mechanism by which the gut microbiota affects distant organs, such as the lungs, is likely partly through microbiota-derived metabolites. (Cait et al. 2018)

Several studies have shown that children with asthma have differentially regulated metabolites and metabolic pathways (Turi et al. 2018; Chiu et al. 2018; Berdnikovs et al. 2022). Metabolites are not just end products, but also function to modulate host biological processes by chemically modifying the upstream pathways (Rinschen et al. 2019). In addition to being endogenously produced by the host, metabolites can be derived from host microbiota, as well as transformed by the gut microbes from xenobiotics, dietary, and other exogenous sources (Johnson et al. 2012; Lee and Hase 2014). Prominent examples include production of SCFAs such as acetate, propionate, and butyrate through bacterial fermentation of dietary fiber, primarily by members of the Firmicutes phylum such as Faecalibacterium, Roseburia, Eubacterium, and Lachnospira (Sasaki et al. 2024).Given that host-microbiome interactions are bidirectional, gut microbiome-derived metabolites could potentially interact with the host’s upstream molecular pathways, and reciprocally, host-derived metabolites could shape the microbial ecology of the gut. Thus, investigating the interrelationship between gut microbiota and metabolites is crucial to understanding the functional role of the gut microbiota in respiratory diseases. In this regard, computational integration of gut-microbiome and metabolome data may help us understand gut microbial ecology and hypothesize functional roles in childhood asthma pathogenesis.

This exploratory study aimed to integrate early-life gut microbiome and metabolome data to identify candidate microbiome–metabolite signatures associated with childhood asthma risk. These findings are intended to generate hypotheses regarding potential biological mechanisms underlying early-life microbial and metabolic influences on asthma development. As a large proportion of gut metabolites enter the circulation and are eventually cleared by the kidneys, (Meyer and Hostetter 2012) we integrated gut microbiome data with metabolome data from both infant urine and stool, measured at the same time point.

## Methods

### Study population

This study was conducted using a subsample of the Infant Susceptibility to Pulmonary Infections and Asthma following Respiratory Syncytial Virus Exposure (INSPIRE), a birth cohort of infants enrolled in the southeastern United States from 2012 to 2014 (see Table E1 for INSPIRE cohort characteristics). Among 1946 infants enrolled in the INSPIRE cohort, 647 infants had gut microbiome data, 264 had urinary metabolome data, and 80 had stool metabolome data. The number of children with both gut microbiome and urinary metabolome data was 124, and the number of children with both gut microbiome and stool metabolome data was 80. Once the missing outcome (current asthma at age 6 years) was accounted for, the number of children with both gut-microbiome data and urinary metabolome data was 89, and the number of children with both gut microbiome data and stool metabolome data was 58. One parent of each participant provided written informed consent for participation in the INSPIRE study. The protocol and informed consent documents were approved by the Institutional Review Board of Vanderbilt University Medical Center (IRB approval number 111299).

### Study design

This study integrates data from children with available metabolome and gut microbiome data. Selection of children for measurement of urine and stool metabolome data was based on a case–control study design that was enriched for wheeze or allergic sensitization during the first year of life and a comparator group of infants who never wheezed and were not sensitized to aeroallergens or food during the first year of life. In the case–control study for metabolome data, the wheeze or allergic sensitization cases and comparator groups were matched on sex and age in months at the time of sample collection.

### Asthma outcome

Childhood asthma at age 6 years was defined as 1) physician-diagnosed or parent-reported physician diagnosis of asthma or use of asthma medications at age 6 years, and 2) any of the following in the 12 months before age 6 years: asthma symptoms, asthma-related systemic steroid use, or acute health care utilization for asthma.

### Gut microbiome data

The method for generating the gut microbiome data and all processing steps has been described in a previous publication (Rosas-Salazar et al. 2021, 2022). In brief, bacterial DNA was extracted from the stool samples using the DNeasy PowerSoil Kit (Qiagen). The hypervariable V4 region of the 16S ribosomal RNA (rRNA) gene was amplified to create sequencing libraries, as described in Kozich et al. 2013 (Kozich et al. 2013). The libraries were sequenced on an Illumina MiSeq platform with 2×250 bp reads. Extraction and PCR negative controls and positive controls with a known mock community (ZymoBIOMICS Microbial Community DNA Standard) were included in all processing steps. The 16S rRNA sequences were processed using the R dada2 package (Callahan et al. 2016a) by following its standard operating procedure. Subsequently, sequences were grouped into amplicon sequence variants (ASVs) and taxonomy was assigned using the SILVA reference database v138 (Quast et al. 2012). Low-quality sequences, chimeras, and non-bacterial sequences were discarded as part of the dada2 pipeline (Callahan et al. 2016b). Potential contaminants were removed using the R decontam package (Davis et al. 2018). We also discarded participant samples with <1,000 sequences (n=10 samples). Mean and median reading depth of each sample are provided in the supplementary CSV file (Repository Data File 1). Mean and median reading depth of each sequence and taxonomic annotation are provided in the supplementary CSV file (Repository Data File 2).

### Metabolome data

The method for generating the urine and stool metabolome has been previously published (Turi et al. 2021). The infant urine samples were collected using an external bag and were immediately transferred to specimen cups and stored at 4 °C. Urine samples were then aliquoted into 2 mL aliquots and stored at −80 °C until shipment for analysis without an interim freeze–thaw cycle. Stool samples were collected from diapers and then transferred to biospecimen cups and stored at 4 °C. Stool samples were then aliquoted into 2 mL aliquots and stored at −80 °C until shipment for analysis without an interim freeze–thaw cycle. Urine and stool samples from the INSPIRE cohort were shipped and prepared for analysis using Metabolon’s (Metabolon Inc., Morrisville, NC) standard solvent extraction method. The extracted samples were divided into equal parts for untargeted analysis on ultra-high-performance liquid chromatography-tandem mass spectrometry (UPLC-MS/MS; positive ionization), UPLC-MS/MS (negative ionization), and UPLC-MS/MS polar platform (negative ionization) (Evans et al. 2009, 2014). Combining these platforms helped us cover a wide range of metabolites, which would not be possible with a single platform (Guo et al. 2015; Shin et al. 2014). In addition, several technical replicate samples were created from a homogeneous pool, containing a small amount of all study samples. The identification of known chemical entities was based on a comparison with metabolomics library entries of purified standards at Metabolon Inc. Additional mass spectral entries were created for biochemicals that were identified by their recurrent nature (both chromatographic and mass spectral) and classical structural analysis but were not confirmed with purified standards. Metabolite relative abundance was defined as its liquid chromatography (LC)-mass spectrometry (MS)-determined peak area, which is proportional to concentration (Kenar et al. 2014). Information related to biochemical pathways and chemical properties was identified from publicly available databases. (DeHaven et al. 2010)

### Statistical methods

For microbiome data, our initial step involved excluding ASVs with more than 90% zeros. Following this filtering process, we retained 55 ASVs in our dataset. To ensure that the observed differences in microbial relative abundances were not solely driven by variations in sequencing depth or compositional effects, we performed normalization using the median of ratios method in the DESeq2 package. For metabolome data, we quantile normalized, log-transformed, Pareto scaled, and imputed missing values with one-half of the minimum observed value for each metabolite if the missing value was less than 25%.

We used a nested cross-validation framework with stratified 5-fold outer cross-validation, 10 repeats of 5-fold inner cross-validation, and sparse partial least squares discriminant analysis (sPLS-DA) in the mixOmics (Rohart et al. 2017) R library to discriminate between children with and without asthma at 6 years of age. All model-building steps, including metabolite feature selection and optimization of model parameters, were performed exclusively within the training folds of each iteration. The held-out validation fold was used only for evaluating model performance, thereby minimizing information leakage between the training and testing datasets. The number of latent components and the number of selected features were optimized within the inner training loop based on the balanced error rate. The feature selection of each component was governed by the sparsity parameters. We used grid search, evaluating a range of numbers to determine the number of features retained (5, 10, 20, and 30). The contribution of microbiome taxa and metabolome compounds to discrimination between phenotypes was calculated by taking the weighted sum of the squared loadings of each variable across the optimal number of sPLS-DA components. The loadings of each variable were weighted by the amount of covariance explained by each sPLS-DA component before taking the sum. The approach of summarizing variable importance across components by aggregating squared loading weights reflects both magnitude and stability of contributions in the multivariate model. Finally, AUC and BER were calculated separately for each outer validation fold. Model performance was evaluated using the predicted class probabilities from the outer validation folds. Confidence intervals for the area under the receiver operating characteristic curve (AUC) were estimated using 2000 bootstrap resampling.

We then used Spearman’s rank correlation to evaluate relationships between prioritized microbiome taxa (genera and species) and metabolites. Spearman's rank correlations were calculated using pairwise complete observations. Numeric zero values were retained as observed values and were not treated as missing. Tied values were assigned average ranks, and two-sided asymptotic p-values were calculated with exact = FALSE. Raw p-values were adjusted using the Benjamini–Hochberg false discovery rate (FDR) method. Correlations with a Bonferroni-adjusted p < 0.05 were considered statistically significant.

Metabolites set over-representation analysis (ORA) and taxon set enrichment analysis (TSEA) for the short-listed metabolome compounds and microbiome genera and species sets were performed in Metaboanalyst (Pang et al. 2021) version 6 and Microbiomeanalyst (Lu et al. 2023) version 2, respectively. The background set for metabolites enrichment analysis was 139 KEGG pathways integrating human (host) and gut bacterial metabolism. Statistical test for ORA of metabolites was performed using a global test, which uses a generalized linear model to compute a ‘Q-stat’ for each metabolite set. The Q-stat is calculated as the average of the Q values calculated for every single metabolite, while the Q value is the squared covariance between the metabolite and the outcome. Statistical tests for TSEA were performed using the hypergeometric test to evaluate whether a particular genus and species set was represented more than expected by chance within the given compound list. One-tailed p-values were provided after adjusting for multiple tests. Throughout the analysis, the Bonferroni multiplicity correction method was applied to the p-values. Finally, we conducted microbial and host source confirmation analysis for the metabolites significantly correlated with microbiome taxa (genera and species) using MetOrgin (Yu et al. 2024) to see agreement with our results.

## Results

### Characteristics of the study population

Of the 1,946 children in the INSPIRE cohort, 59.9% (n=1,166) had 6-year follow-up to ascertain childhood asthma. Among the 1,166 children, 18.5% (n=216) were diagnosed with asthma at age 6 years. Of those with asthma, 61.1% (n=132) were male, 63.0% (n=136) were white, 19.0% (n=41) were black, 6.0% (n=13) were Hispanic, and 12.0% (n=26) were other races or multi-race or unknown (Table 1). Of the 1,166 participants, 58 had stool metabolome data, 199 had urine metabolome data, and 402 had stool microbiome data available (see Table E1 for details). The number of children with overlapping gut microbiome and stool metabolome data was 58, and the number of children with overlapping gut microbiome and urine metabolome data was 89 (Figure E1).

**Table 1:** Study participant demographics, other characteristics, and data availability for multi-omics analysis by asthma outcome

	Current Asthma at 6-Years of Age			
	Yes (N=216)	No (N=950)	Missing (N=780)	Overall (N=1946)
Child sex				
Male	132 (61.1%)	467 (49.2%)	421 (54.0%)	1020 (52.4%)
Female	84 (38.9%)	483 (50.8%)	359 (46.0%)	926 (47.6%)
Child age at enrollment in days				
Mean (SD)	64.5 (52.6)	57.0 (50.9)	61.5 (51.9)	59.6 (51.5)
Median [Min, Max]	62.0 [2.00, 250]	49.5 [3.00, 258]	58.0 [3.00, 244]	55.0 [2.00, 258]
Child race and ethnicity				
White (non-Hispanic)	136 (63.0%)	640 (67.4%)	491 (62.9%)	1267 (65.1%)
Black (non-Hispanic)	41 (19.0%)	151 (15.9%)	151 (19.4%)	343 (17.6%)
Hispanic	13 (6.0%)	90 (9.5%)	67 (8.6%)	170 (8.7%)
Other/multi/unkown	26 (12.0%)	69 (7.3%)	71 (9.1%)	166 (8.5%)
Secondhand smoke exposure				
Exposed	100 (46.3%)	368 (38.7%)	443 (56.8%)	911 (46.8%)
Not exposed	116 (53.7%)	581 (61.2%)	337 (43.2%)	1034 (53.1%)
Missing	0 (0%)	1 (0.1%)	0 (0%)	1 (0.1%)
Any breastfeeding				
Yes	164 (75.9%)	782 (82.3%)	537 (68.8%)	1483 (76.2%)
No	52 (24.1%)	168 (17.7%)	243 (31.2%)	463 (23.8%)
Maternal asthma				
Yes	78 (36.1%)	153 (16.1%)	148 (19.0%)	379 (19.5%)
No	138 (63.9%)	796 (83.8%)	632 (81.0%)	1566 (80.5%)
Missing	0 (0%)	1 (0.1%)	0 (0%)	1 (0.1%)
Urine metabolites data				
Yes	50 (23.1%)	149 (15.7%)	65 (8.3%)	264 (13.6%)
No	166 (76.9%)	801 (84.3%)	715 (91.7%)	1682 (86.4%)
Stool metabolites data				
Yes	17 (7.9%)	41 (4.3%)	22 (2.8%)	80 (4.1%)
No	199 (92.1%)	909 (95.7%)	758 (97.2%)	1866 (95.9%)
Stool microbiome data				
Yes	73 (33.8%)	329 (34.6%)	245 (31.4%)	647 (33.2%)
No	143 (66.2%)	621 (65.4%)	535 (68.6%)	1299 (66.8%)

### Prioritizing gut-microbiome taxa and metabolome compounds relevant to childhood asthma

**Fig. 1:**
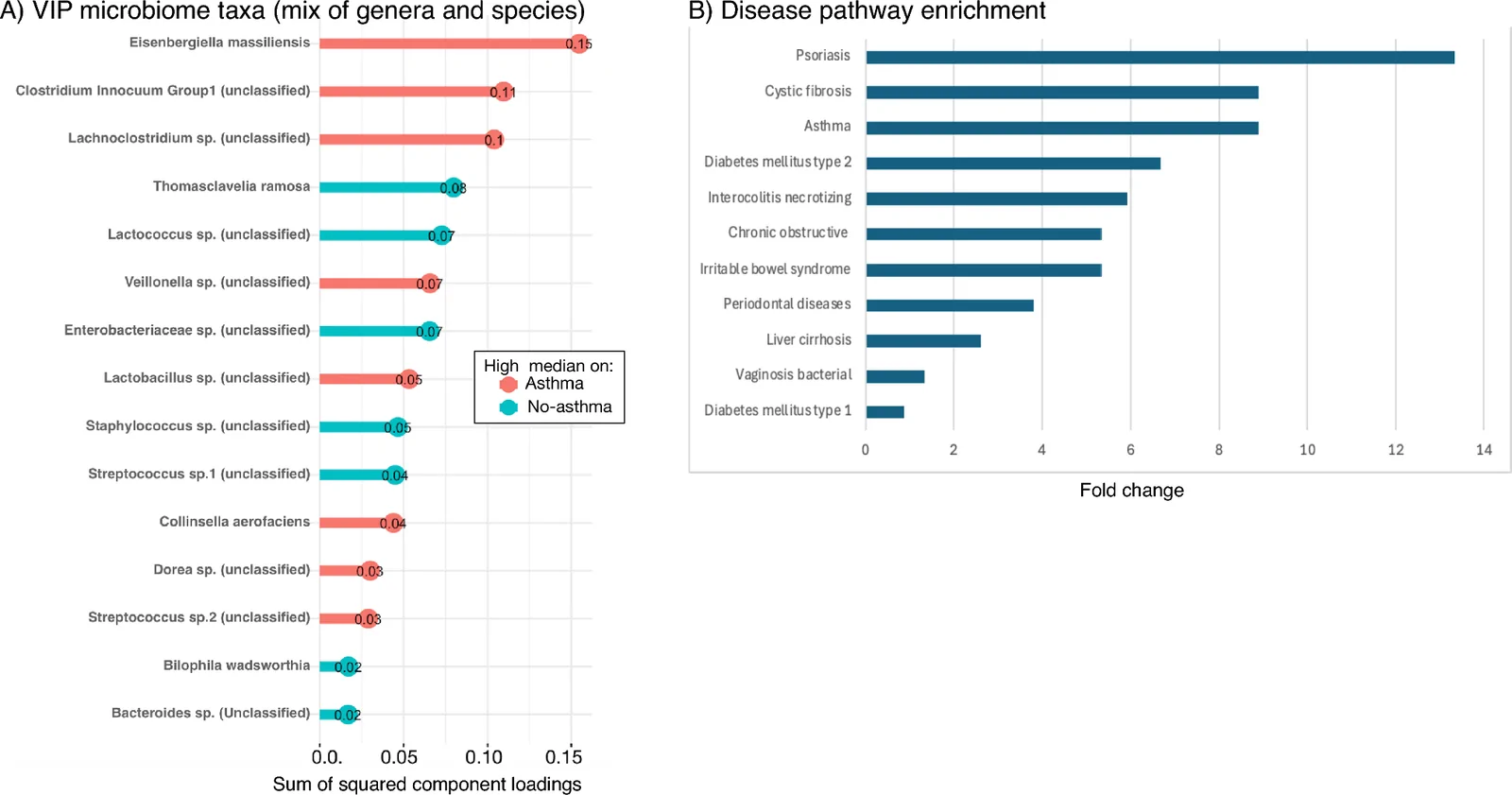
**A** Top 15 very important (VIP) gut microbiome taxa (mix of genera and species) discriminating between 6-year asthma and non-asthma using sparse Partial Least Squares Discriminant Analysis (sPLS-DA); **B** Enrichment analysis of top VIP gut microbiome genera and species discriminating between 6-year asthma and non-Asthma

We used nested sPLS-DA to separately prioritize microbiome taxa (genera and species) and metabolites that discriminate asthma vs. no asthma at age 6 years (Figure E2, Table E2, and Table E3). The discriminating performance of the gut microbiome based on the first two sPLS-DA components had a mean AUC of 0.55 (95% CI = [0.50, 0.60]) and a mean balanced error rate (BER) of 0.49 (95% CI[0.0.48, 0.50]). The fifteen most important genera and species of the microbiome in discriminating between asthma and non-asthma children were *Eisenbergiella massiliensis*, *Clostridium Innocuum* group 1, *Lachnoclostridium* sp. (Unclassified), *Thomasclavelia ramosum*, *Lactococcus* sp. (Unclassified), *Veillonella* sp. (Unclassified), *Enterobacteriaceae* sp.* (Unclassified), Lactobacillus* sp. (Unclassified), *Staphylococcus* sp. (Unclassified), *Streptococcus* sp.*1* (Unclassified), *Collinsella aerofaciens, Dorea* sp. (Unclassified), *Streptococcus* sp.*2* (Unclassified), *Bilophila Wadsworthia, and Bacteroides* sp.* (Unclassified)* (Fig. 1A and Table E4)*.* The enrichment analysis of the 15 microbiome genus and species set shows that they were enriched for microbiomes previously shown to be associated with asthma, Cystic fibrosis, diabetes, necrotizing colitis, and irritable bowel syndrome (Fig. 1B and Table E5). However, most disease-pathway terms did not remain statistically significant after multiple-testing correction and are presented solely as descriptive findings.

**Fig. 2:**
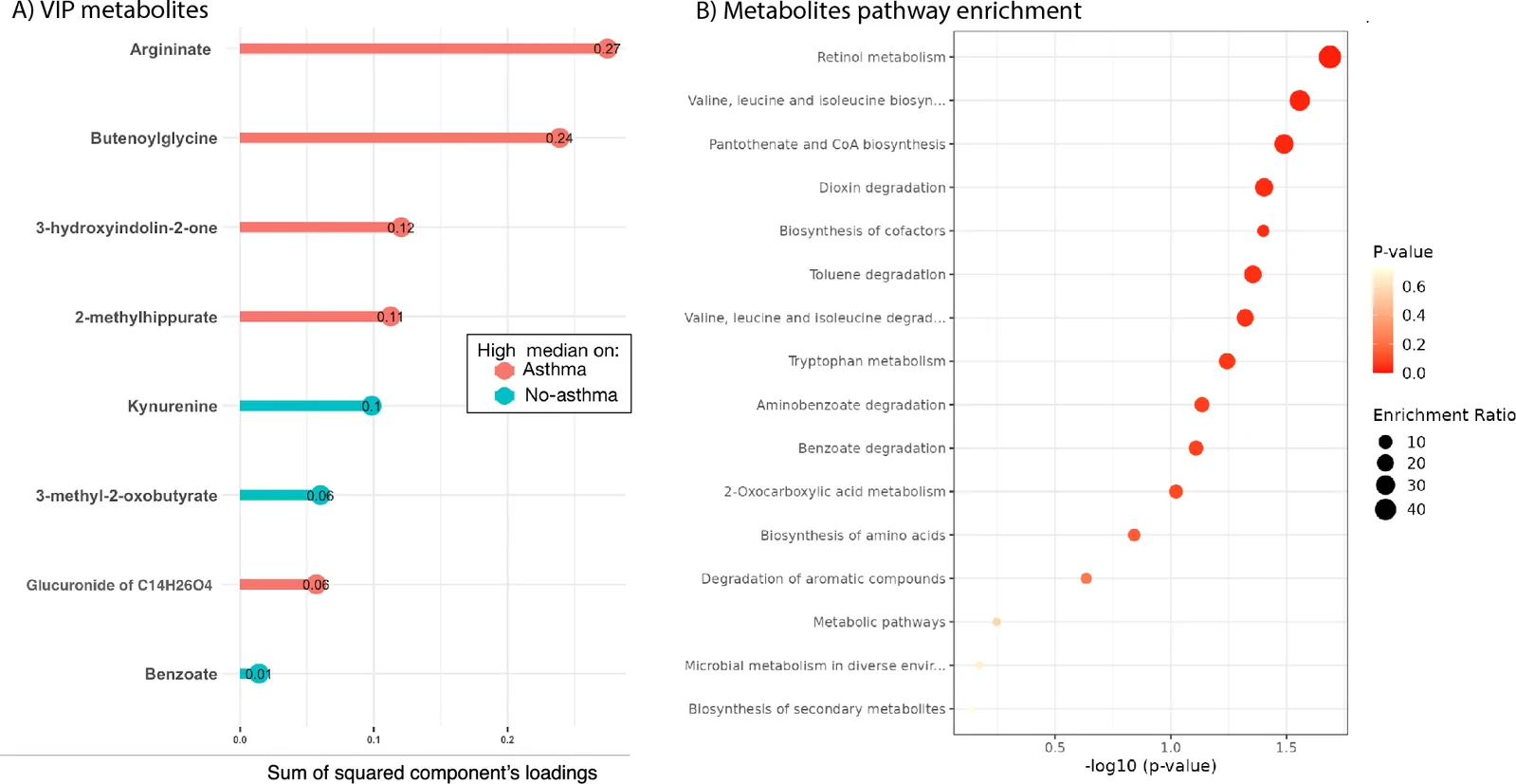
**A** Top very important (VIP) urine metabolites discriminating between 6-year asthma and non-asthma using sparse Partial Least Squares Discriminant Analysis (sPLS-DA); and **B** Enrichment analysis of top VIP urine metabolites discriminating between 6-year asthma and non-Asthma. Present p-values are nominal, and pathways were insignificant after FDR adjustment

The discriminating performance of urine metabolome data based on the first two components of PLS-DA had a mean AUC of 0.61 (95% CI[0.53, 0.70]) and mean BER of 0.48 (95% CI[0.0.41, 0.53]). The most important urine metabolites (Fig. 2A and Table E6) in discriminating between those with and without asthma are argininate, butenoylglycine, 3-hydroxyindolin-2-one, 2-methylhippurate, kynurenine, 3-methyl-2-oxobutyrate, glucuronide of C14H26O4 (1), and benzoate. Based on the metabolite list above, enriched metabolic pathways include retinol metabolism; valine, leucine, and isoleucine biosynthesis; pantothenate and CoA biosynthesis; dioxin degradation; biosynthesis of cofactors; toluene degradation; and valine, leucine, and isoleucine degradation (Fig. 2B). However, these pathways were not significant after FDR adjustment.

**Fig. 3:**
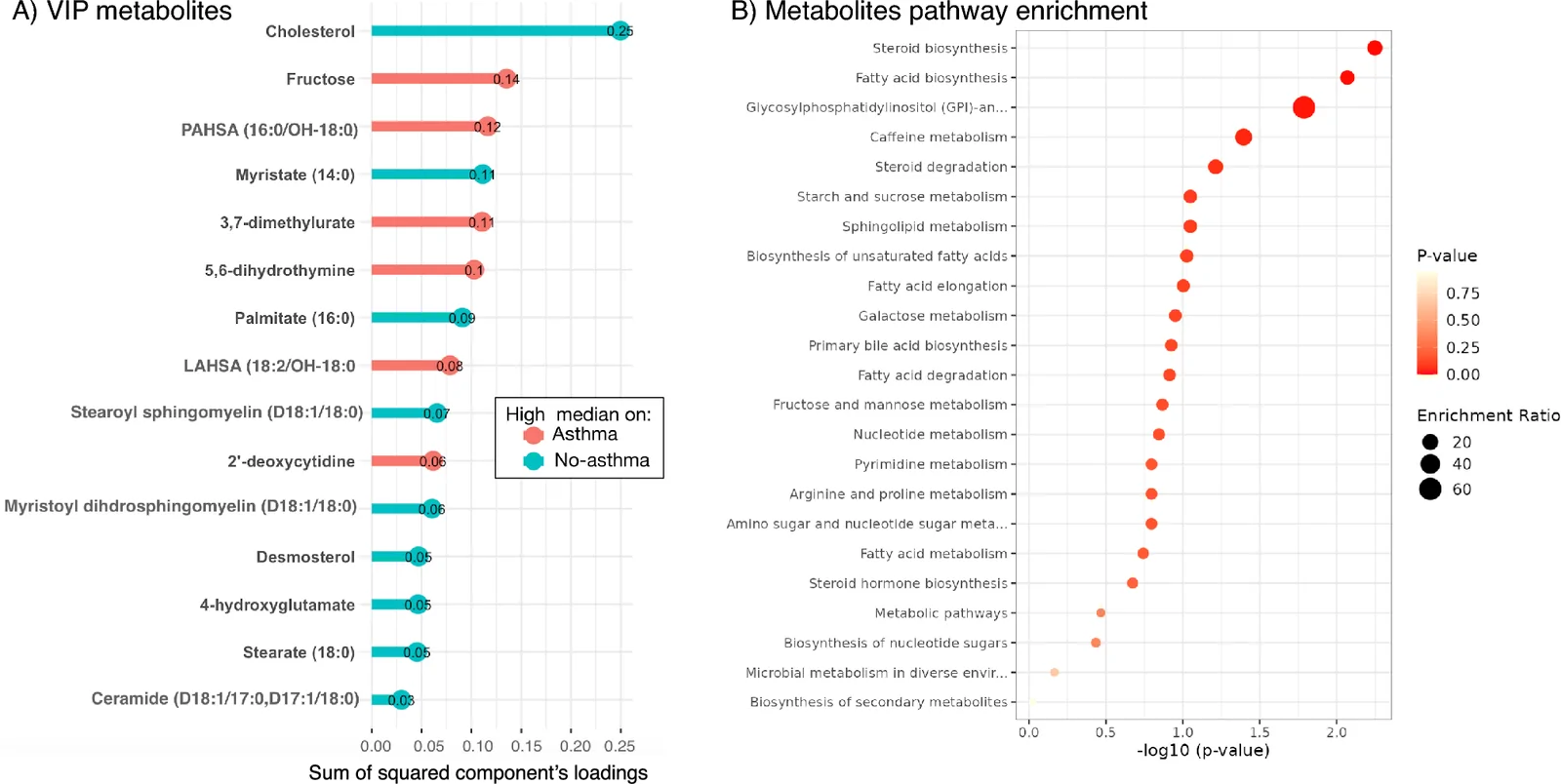
**A** Top very important (VIP) stool metabolites discriminating between 6-year-old asthma and non-asthma diagnosis using sparse Partial Least Squares Discriminant Analysis (sPLS-DA); and **B** Enrichment analysis of top VIP stool metabolites discriminating between 6-year asthma and non-asthma. Present p-values are nominal, and pathways were insignificant after FDR adjustment

The performance of stool metabolome data based on the first two components of sPLS-DA in discriminating asthma outcome had a mean AUC of 0.60 (95% CI[0.55, 0.64]) and mean BER of 0.53 (95% CI[0.0.50, 0.56]). The most important stool metabolites (Fig. 3A and Table E7) in discriminating between those with and without asthma were cholesterol, fructose, palmitic acid ester of hydroxy stearic acid [PAHSA](16:0/OH-18:0), myristate (14:0), 3,7-dimethylurate, 5,6-dihydrothymine, palmitate (16:0), linoleic acid hydroxy stearic acid [LAHSA]( (18:2/OH-18:0), stearoyl sphingomyelin (d18:1/18:0), 2'-deoxycytidine, myristoyl dihydrosphingomyelin (d18:0/14:0), desmosterol, 4-hydroxyglutamate stearate (18:0), and ceramide (d18:1/17:0, d17:1/18:0). Based on the metabolites list above, enriched metabolic pathways include steroid biosynthesis, fatty acid biosynthesis, glycosylphosphatidylinositol (GPI), caffeine metabolism, steroid degradation, starch and sucrose metabolism, and sphingolipid metabolism (Fig. 3B). However, these pathways were not significant after FDR adjustment.

### Correlation between exploratory asthma-relevant gut microbiome taxa and exploratory asthma-relevant metabolome compounds

**Fig. 4:**
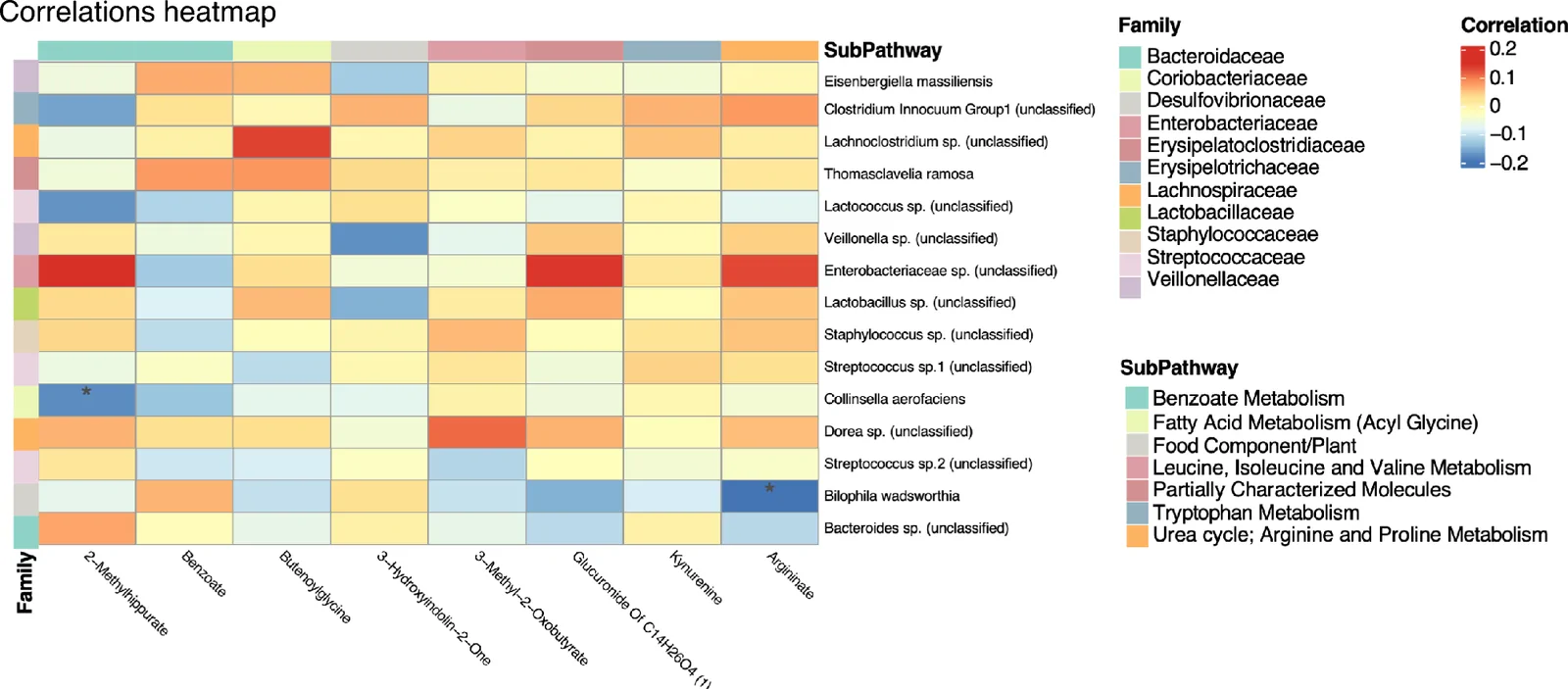
Heatmap of Spearman rank correlation between very important (VIP) microbiome genera and species and VIP urine metabolites discriminating between 6-year-old asthma and non-asthma diagnosis. VIP microbiome taxa (mix of genera and species) and metabolites were identified using sparse Partial Least Squares Discriminant Analysis (sPLS-DA). Correlation significance was determined based on nominal p-value of ≤0.05

**Fig. 5:**
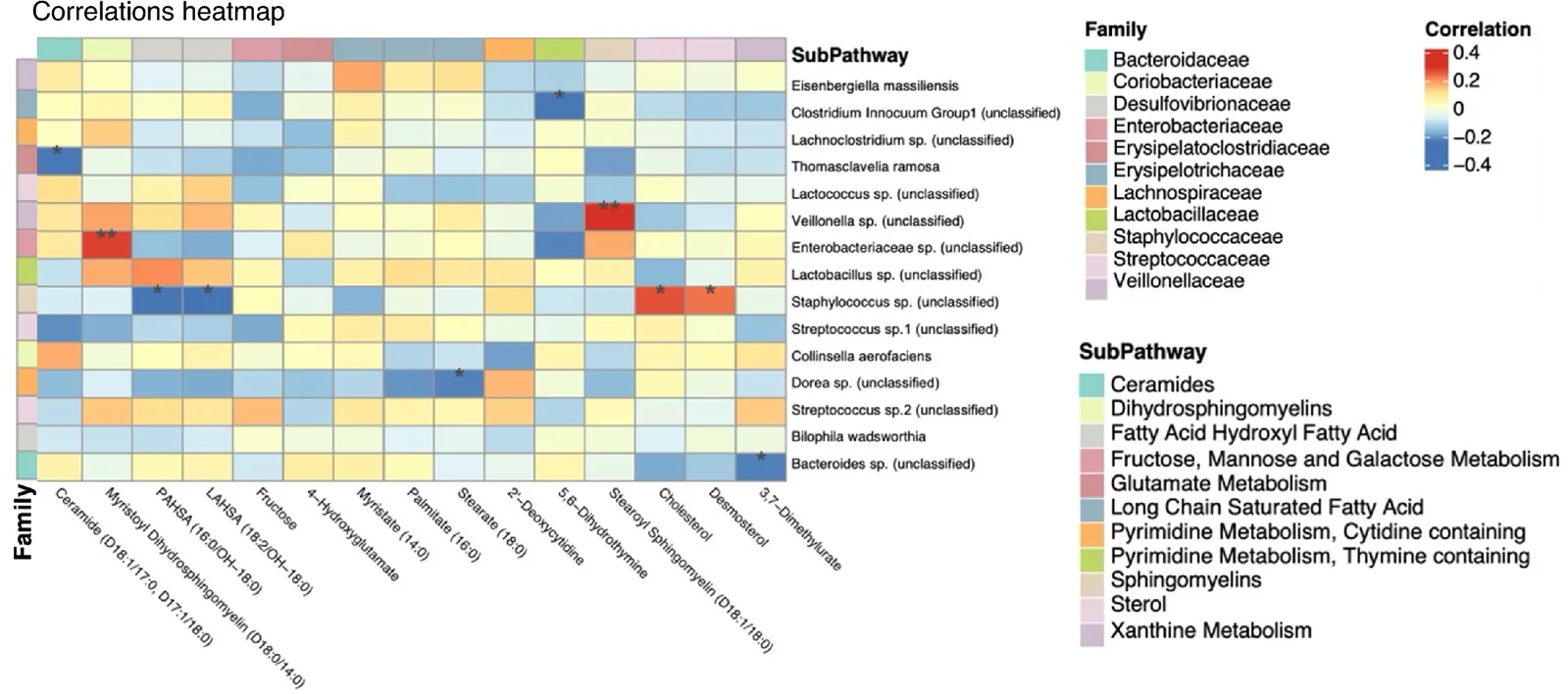
Heatmap of Spearman rank correlation between very important (VIP) microbiome genera and species and VIP stool metabolites discriminating between 6-year-old asthma and non-asthma diagnosis. VIP microbiome texa (mix of genera and species) and metabolites were identified using sparse Partial Least Squares Discriminant Analysis (sPLS-DA). Correlation significance was determined based on nominal p-value of ≤0.05

Finally, we investigated the relationship between the selected childhood exploratory asthma-relevant stool-microbiome genera and species and urine and stool metabolome compounds using Spearman rank correlation (Fig. 4 for urine metabolites and stool microbiome and Fig. 5 for stool metabolites and stool microbiome and Repository file 3 for detailed correlation statistics). Correlations were assessed using a nominal P-value threshold of ≤0.05, with Bonferroni correction applied to account for multiple testing. Although some correlations met the nominal significance threshold, none remained statistically significant after Bonferroni correction. There were two weak but significant correlations between microbiome and urine metabolites. Significant correlations include a negative correlation between *Collinsella aerofaciens* and 2-methylhippurate and a negative correlation between *Bilophila wadsworthia* and argininate.

We next tested for correlations between the stool microbiome and stool metabolites (Fig. 5). Several significant correlations were identified in associations between the stool microbiome and metabolites, primarily involving lipid-related metabolites. *Staphylococcus* sp. (unclassified) was negatively correlated with branched fatty acid esters of hydroxy fatty acids (FAHFAs), including palmitic Acid Ester of Hydroxy Stearic Acid [PAHSA] (16:0/OH-18:0) and Linoleic Acid Hydroxy Stearic Acid [LAHSA] (18:2/OH-18:0), whereas positively correlated with cholesterol and desmosterol. *Enterobacteriaceae* sp. (unclassified) was positively correlated with myristoyl dihydrosphingomyelin and *Veillonella* sp. (unclassified) showed positive correlations with stearoyl sphingomyelin (d18:1/18:0). *Thomasclavelia ramosa* showed negative correlations with ceramide, *Dorea* sp. (unclassified) was negatively correlated with stearate, *Bacteroides* sp.(unclassified) was negatively correlated with 3,7-dimethylurate, and *Clostridium innocuum* group 1 was negatively correlated with 5,6-dihydrothymine.

### MetOrigin assigned putative source categories of metabolites significantly correlatied microbiome taxa

**Fig. 6:**
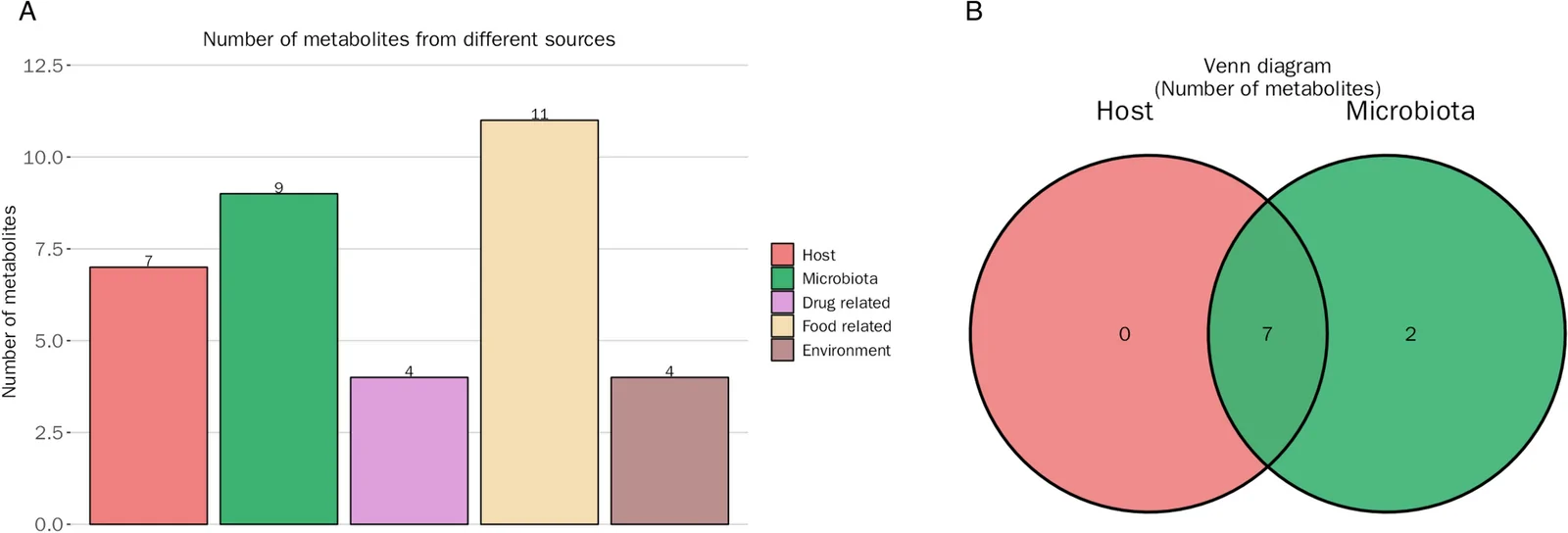
MetOrigin putative source assignment (prediction) for urine and stool metabolites that were significantly correlated with the microbiome taxa (mix of genera and species). A) The source of metabolites can be endogenous from the host, food, drug, microbiome, and/or environment. The assigned source categories are overlapping and therefore sum to more than 11 metabolites. B) Metabolites can be metabolized by the host, microbiome, or co-metabolized by the host and microbiome

To confirm if, in fact, our exploratory prior selected metabolites-microbiome correlation results are supported by previous literature on microbiome metabolites relationships, we conducted metabolites origin enrichment analysis in the MetOrigin database. We started with 11 metabolites (two from urine and 9 from stool) significantly correlated with one or more microbiome taxa. MetOrigin putatively assigns the majority (n=7) of the metabolites that were significantly correlated with microbiome taxa to likely be co-metabolised by host and microbiome, and two were solely metabolised by the microbiome (Fig. 6A and Table E8). Categories in Fig. 6 panel A overlap, and therefore sum to more than the 11 metabolites. Moreover, MetOrigin source enrichment analysis shows that the majority of the metabolites are likely derived from food through microbiome metabolism or co-metabolism with the host (Fig. 6B and Table E8).

## Discussion

This exploratory study integrated early-life gut microbiome and metabolome data to identify candidate microbiome–metabolite signatures associated with childhood asthma risk. The study identified patterns in the infant gut microbiome and metabolome that were associated with the development of asthma at age 6 years. The results from follow-up correlation analysis between top asthma risk-related metabolites and microbiome taxa were intended to generate hypotheses regarding potential biological mechanisms underlying early-life microbial and metabolic influences on asthma development. Our analysis shows that early-life microbiome and metabolome patterns preselected on childhood asthma risk were correlated with each other. We hypothesize that the selected microbiome taxa and metabolites and their correlations were likely influenced by the early-life mode of feeding and microbial colonization trajectories.

A disease pathway analysis showed that the top microbiome genera and species that discriminated between those with and without asthma at age 6 years were enriched in disease pathways previously associated with decreased asthma risk and inflammatory bowel diseases (Zheng et al. 2016; Hilty et al. 2010). Similarly, the top urine and stool metabolites in our analysis were enriched in disease pathways that are known to be relevant to early childhood feeding (Vercelli 2003; Benson et al. 2011). These findings highlight the biological relevance of the prioritized microbiome taxa (genus and species) and metabolites, strengthening confidence in our integrative approach.

Among observed correlations between individual microbiome genera and species and metabolites, the negative correlations between *Staphylococcus* sp. (unclassified) and FAHFAs, including PAHSA (16:0/OH-18:0)and LAHSA (18:2/OH-18:0), together with its positive associations with cholesterol and desmosterol, suggest that early-life gut microbial colonization, dietary exposures, and host lipid metabolism may co-vary within a shared metabolic and ecological milieu. FAHFAs are lipid mediators with endogenous and exogenous sources (including human milk) and are reported to have anti-inflammatory and metabolic regulatory properties, including modulation of immune responses and glucose metabolism (Benlebna et al. 2021; Spann et al. 2012). However, FAHFAs in stool may reflect reduced intestinal uptake, microbial modification, or compensatory responses to chronic immune activation (Lee et al. 2016; Lin et al. 2015). The infant gut microbiome undergoes rapid, exposure-dependent succession in early life, with early dominance of facultative anaerobes, including Staphylococcus, prior to maturation into obligate anaerobe–dominated communities. Previous amplicon studies have identified a core microbial signature of the human breast milk microbiome, dominated primarily by taxa belonging to the Staphylococcus and *Streptococcus* genera (Ferretti et al. 2025). In parallel, cholesterol and desmosterol, which are important for infant growth as regulators of immune cell function, membrane organization, and inflammatory signaling, (Aguilar-Ballester et al. 2020) are high in breast milk-fed infants. It has been reported that cholesterol significantly stimulates the proliferation and growth rate of Staphylococcus species (Shine et al. 1993). The bacteria break down host cholesterol and use it as a metabolic resource to multiply Staphylococcus sp. rapidly, (Delekta et al. 2018; Samanta et al. 2017) which provides indirect evidence that a breast milk-based diet during early infancy may favour such a gut microbiome colonization. Together, these findings support the interpretation that *Staphylococcus* abundance and lipid metabolites, including FAHFAs and sterols, co-vary within an early-life gut ecological and metabolic milieu shaped by feeding exposures, microbial succession, and host lipid metabolism, rather than indicating a direct mechanistic relationship.

Several associations involved sphingolipid metabolism, a pathway increasingly recognized in epithelial barrier function and inflammatory signaling. Our study finds that *Enterobacteriaceae* sp. (unclassified) was positively correlated with myristoyl dihydrosphingomyelin, whereas *Veillonella* sp. (unclassified) was positively associated with stearoyl sphingomyelin (d18:1/18:0). In contrast, *Thomasclavelia ramosa* was negatively correlated with ceramide, suggesting an inverse relationship between this taxon and sphingolipid abundance. Enterobacteriaceae and *Veillonella* sp. are dominant pioneer colonizers of the newborn gut (Bittinger et al. 2020; Vera et al. 2026). *Thomasclavelia ramosa* has been linked to host lipid metabolism and immune homeostasis in previous studies. Simultaneously, sphingomyelins are among the most abundant lipids found in human breast milk and infant formula (Pantazi et al. 2023; Yuan et al. 2024; Wu et al. 2023). Sphingomyelins and related ceramide metabolites participate in cellular membrane dynamics, immune activation, and epithelial responses to environmental stressors (Hannun and Obeid 2018). As infants likely consume a milk-based diet at this age, there will naturally be a high abundance of myristoyl dihydrosphingomyelin in the gut lumen (Merrill 2025). Enterobacteriaceae thrive during this milk-dominant stage of development; (Gopalakrishna and Hand 2020) a positive correlation may reflect the shared timeline of a milk-rich diet and early colonizing microbiome genera. These findings are consistent with previous childhood asthma microbiome studies that have identified relationships between microbial composition and metabolite profiles, (Lee-Sarwar et al. 2022) although specific lipid pathways remain incompletely characterized.

Beyond lipid metabolism,*Bacteroides* sp. (Unclassified) was negatively correlated with 3,7-dimethylurate, indicating that microbial associations extended to purine metabolism. *Bacteroides* sp. thrive on human milk oligosaccharides (HMOs) found in breast milk. *Bacteroides* sp. levels often signify a breastfed gut environment (Walsh et al. 2020). Conversely, 3,7-dimethylurate is a byproduct of xanthine/caffeine-like compounds (Monteiro et al. 2016). In infants, xanthine compound typically enters their system via maternal diet (caffeine passing through breast milk) or specific ingredients in infant formulas (Blake et al. 2006; Wang et al. 2025). Infants born vaginally naturally acquire significantly more *Bacteroides* sp. at birth than those born via C-Section (Coelho et al. 2021) This initial bacterial baseline alters how the infant digests and metabolizes early dietary inputs, including caffeine passing through breast milk or from formula (Wang et al. 2025; Matharu et al. 2022). The negative correlation suggests that a high abundance of Bacteroides may alter how the infant's body or gut microbes process these xanthine compounds, (Pan et al. 2025) leading to faster clearance or lower accumulation of 3,7-dimethylurate*.*

The strength of our study lies in its integration of early life microbiome-metabolome data from a birth cohort that has been longitudinally followed and characterized for asthma outcomes. Integrating gut microbiome data with metabolome data representing both the gut microbiome and metabolome (as measured in both stool and urine) allowed us to uncover microbiome-metabolome relationships that have local and systemic effects. In addition, we used several statistical approaches that account for high-dimensional and correlated data to extract patterns relevant to the clinical outcomes. There are limitations to this study. First, the metabolomics subset was enriched for early wheeze and allergy sensitization phenotypes; the reported discrimination metrics may reflect both asthma-related biology and the biological features associated with early wheeze/allergic sensitization. Thus, these performance estimates should not be interpreted as directly representing predictive performance in the general population. Rather, they represent discrimination within an enriched high-risk sample designed to maximize the identification of early-life metabolic signatures associated with subsequent asthma development. Second, as the results are computationally derived associations or correlations, we do not claim directionality or causality between microbiome and metabolites. Third, we used microbiome data from 16S ribosomal RNA (rRNA), which only sequences a specific one variable region of the 16S rRNA gene (not the entire region or gene), and it does not identify microorganisms at a species level, constraining interpretation to higher taxonomic levels whenever species-level identification was not available. Forth, stool-based microbiome profiling may not fully capture the complexity of the gut microbiome. Specifically, stool samples primarily reflect luminal microbial communities and may not adequately represent mucosa-associated microbes or spatial heterogeneity across different regions of the gastrointestinal tract. Fifth, given the small sample size of the stool metabolome subset (n = 58), even nested cross-validation may yield unstable estimates of model performance; therefore, these results are interpreted as exploratory. Sixth, no individual dietary-intake data were analyzed in this study. Consequently, discussions about infant dietary exposures are merely speculative explanations from prior literature, not as findings from this cohort.

In conclusion, by integrating the infant gut microbiome and metabolome, we uncovered novel correlations that are relevant to asthma development. We identified correlations between specific microbiome genera and species and metabolic compounds that we hypothesize are involved in the pathobiology of childhood asthma. We acknowledge that confirmation requires replication or experimental studies; however, these results may inform experimentally testable hypotheses. Moreover, most of the metabolic compounds that were important in discriminating between asthma and non-asthma groups were generated from food sources metabolized by microbiota, suggesting that early life diet is important in gut microbial ecology and the production of metabolites relevant to early life child respiratory health, including asthma development.

## Supplementary Information

Below is the link to the electronic supplementary material.

Supplementary file1 (PDF 23 KB)—Flow chart showing analysis process and corresponding sample size at each analysis step

Supplementary file2 (PDF 105 KB)—Partial Least Squares Discriminant Analysis (sPLS-DA) performance in discriminating between 6-year-old asthma and non-asthma diagnosis based on stool microbiome, urine metabolome, and stool metabolome data

Supplementary file3 (CSV 48 KB)

Supplementary file4 (DOCX 34 KB)

Supplementary file5 (CSV 19 KB)

Supplementary file6 (CSV 10 KB)

## Data Availability

Data is available from the senior author, Tina Hartert, with a reasonable request.
