# Supplementary material for: Exploratory integrative analysis of infant gut microbiome and metabolome suggests diet- and microbiome-derived metabolites associated with childhood asthma
Source: Metabolomics. 2026 Sep 27;22(5):162. doi: 10.1007/s11306-026-02540-3 (PMC13616850; doi:10.1007/s11306-026-02540-3)
Supplement: Supplementary file 1 [file 11306_2026_2540_MOESM1_ESM.pdf]

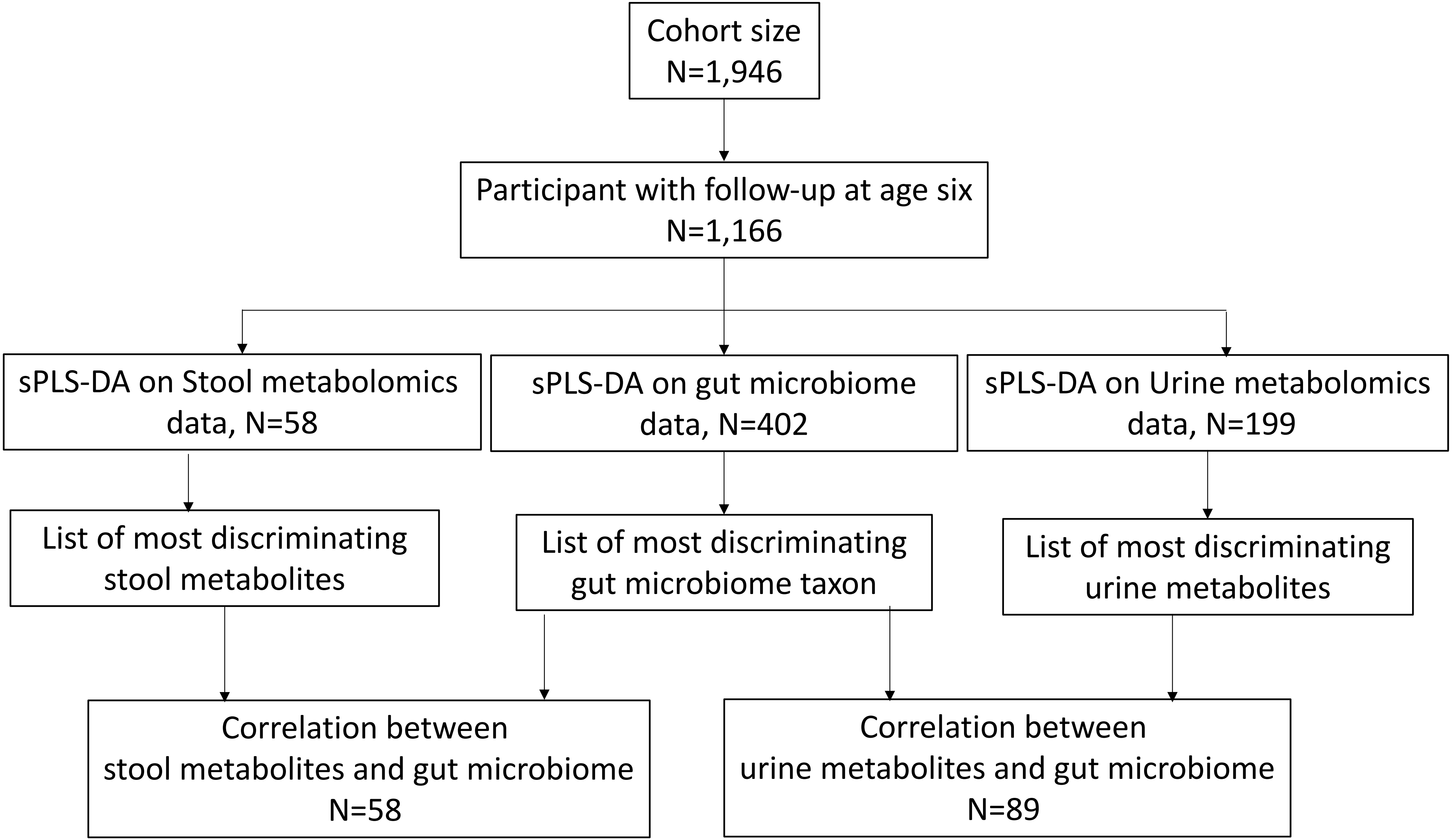
Cohort size
N=1,946
Participant with follow-up at age six
N=1,166
sPLS-DA on Stool metabolomics data, N=58
sPLS-DA on gut microbiome data, N=402
sPLS-DA on Urine metabolomics data, N=199
List of most discriminating stool metabolites
List of most discriminating gut microbiome taxon
List of most discriminating urine metabolites
Correlation between stool metabolites and gut microbiome
N=58
Correlation between urine metabolites and gut microbiome
N=89
