# Supplementary material for: Exploratory integrative analysis of infant gut microbiome and metabolome suggests diet- and microbiome-derived metabolites associated with childhood asthma
Source: Metabolomics. 2026 Sep 27;22(5):162. doi: 10.1007/s11306-026-02540-3 (PMC13616850; doi:10.1007/s11306-026-02540-3)
Supplement: Supplementary file 2 [file 11306_2026_2540_MOESM2_ESM.pdf]

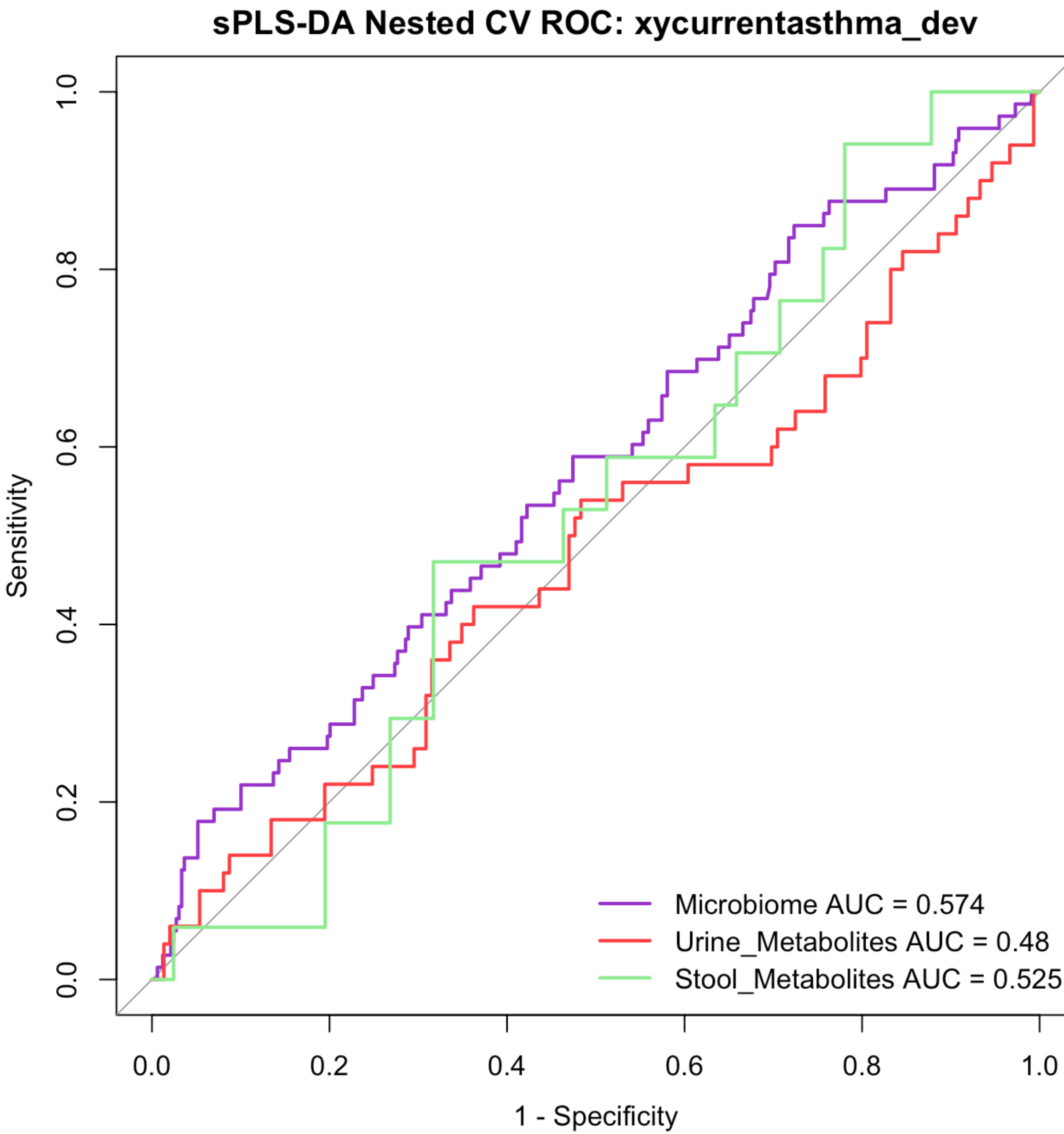

sPLS-DA Nested CV ROC: xycurrentasthma_dev
Sensitivity
0.0
0.2
0.4
0.6
0.8
1.0
1 - Specificity
Microbiome AUC = 0.574
Urine_Metabolites AUC = 0.48
Stool_Metabolites AUC = 0.525
