# Supplementary material for: Exploratory integrative analysis of infant gut microbiome and metabolome suggests diet- and microbiome-derived metabolites associated with childhood asthma
Source: Metabolomics. 2026 Sep 27;22(5):162. doi: 10.1007/s11306-026-02540-3 (PMC13616850; doi:10.1007/s11306-026-02540-3)
Supplement: Supplementary file 3 [file 11306_2026_2540_MOESM4_ESM.docx]

**Supplementary Tables**

**Exploratory Integrative Analysis of Infant Gut Microbiome and Metabolome Suggests Diet- and microbiome-derived Metabolites Associated with Childhood Asthma**

Kedir N Turi^1^, Xiliu Wu^1^, Yajing Li^2^, Christian Rosas-Salazar^3^, Tebeb Gebretsadik^4^, Meghan H. Shilts^5^, James Gern^6^, Yaomin Xu^2^, and Tina V Hartert^3,5^

^1^Department of Epidemiology and Biostatistics, Indiana University, Bloomington, IN

^2^Department of Health Data Science & Biostatistics, Peter O’Donnell Jr. School of Public Health, UT Southwestern Medical Center

^3^Department of Pediatrics, Vanderbilt University Medical Center, Nashville, TN

^4^Department of Biostatistics, Vanderbilt University Medical Center, Nashville, TN

^5^Department of Medicine; Vanderbilt University Medical Center, Nashville, TN

^6^Department of Pediatrics, University of Wisconsin, Madison, WI 53705, USA

Table E1. Study participant demographic and asthma outcomes characteristics by data availability for multi-omics analysis.

|  | **Total**  (N=1946) | **Urine metabolome data available** | | **Stool metabolome data available** | | **Stool microbiome data available** | |
| --- | --- | --- | --- | --- | --- | --- | --- |
|  |  | Yes  (N=264) | No  (N=1682) | Yes  (N=80) | No  (N=1866) | Yes  (N=647) | No  (N=1299) |
| Sex |  |  |  |  |  |  |  |
| Male | 1020 (52.4%) | 164 (62.1%) | 856 (50.9%) | 48 (60.0%) | 972 (52.1%) | 348 (53.8%) | 672 (51.7%) |
| Female | 926 (47.6%) | 100 (37.9%) | 826 (49.1%) | 32 (40.0%) | 894 (47.9%) | 299 (46.2%) | 627 (48.3%) |
| Race |  |  |  |  |  |  |  |
| White | 1267 (65.1%) | 165 (62.5%) | 1102 (65.5%) | 40 (50.0%) | 1227 (65.8%) | 417 (64.5%) | 850 (65.4%) |
| Black | 343 (17.6%) | 53 (20.1%) | 290 (17.2%) | 19 (23.8%) | 324 (17.4%) | 114 (17.6%) | 229 (17.6%) |
| Hispanic | 170 (8.7%) | 27 (10.2%) | 143 (8.5%) | 10 (12.5%) | 160 (8.6%) | 61 (9.4%) | 109 (8.4%) |
| Other/multi/unknown | 166 (8.5%) | 19 (7.2%) | 147 (8.7%) | 11 (13.8%) | 155 (8.3%) | 55 (8.5%) | 111 (8.5%) |
| Secondhand Smoking | | | | | | | |
| Exposed | 911 (46.8%) | 100 (37.9%) | 811 (48.2%) | 38 (47.5%) | 873 (46.8%) | 321 (49.6%) | 590 (45.4%) |
| Not exposed | 1034 (53.1%) | 164 (62.1%) | 870 (51.7%) | 42 (52.5%) | 992 (53.2%) | 326 (50.4%) | 708 (54.5%) |
| Data Not Available | 1 (0.1%) | 0 (0%) | 1 (0.1%) | 0 (0%) | 1 (0.1%) | 0 (0%) | 1 (0.1%) |
| Breast Feeding |  |  |  |  |  |  |  |
| No | 463 (23.8%) | 45 (17.0%) | 418 (24.9%) | 18 (22.5%) | 445 (23.8%) | 137 (21.2%) | 326 (25.1%) |
| Yes | 1483 (76.2%) | 219 (83.0%) | 1264 (75.1%) | 62 (77.5%) | 1421 (76.2%) | 510 (78.8%) | 973 (74.9%) |
| Age (Days) at biospecimen sampling | |  |  |  |  |  |  |
| Mean (SD) | 59.6 (51.5) | 55.9 (46.4) | 60.2 (52.3) | 56.6 (50.5) | 59.8 (51.6) | 54.5 (52.5) | 62.2 (50.8) |
| Median [Min, Max] | 55.0 [2.00, 258] | 58.0 [2.00, 206] | 55.0 [3.00, 258] | 56.5 [2.00, 185] | 55.0 [2.00, 258] | 34.0 [2.00, 257] | 61.0 [3.00, 258] |
| Asthma at 6 years |  |  |  |  |  |  |  |
| Yes | 216 (11.1%) | 50 (18.9%) | 166 (9.9%) | 17 (21.3%) | 199 (10.7%) | 73 (11.3%) | 143 (11.0%) |
| No | 950 (48.8%) | 149 (56.4%) | 801 (47.6%) | 41 (51.3%) | 909 (48.7%) | 329 (50.9%) | 621 (47.8%) |
| Data Not Available | 780 (40.1%) | 65 (24.6%) | 715 (42.5%) | 22 (27.5%) | 758 (40.6%) | 245 (37.9%) | 535 (41.2%) |

Table E2. Sparse Partial Least Squares Discriminant Analysis (sPLS-DA) performance in discriminating asthma and non-asthma diagnosis at age six based on stool microbiome, urine metabolome, and stool metabolome data.

| Data | Metric | Mean | 95% Confidence Interval |
| --- | --- | --- | --- |
| Stool microbiome | Accuracy | 0.8134259 | [0.8059259, 0.8189167] |
|  | BER | 0.4927739 | [0.4809557, 0.5030303] |
|  | AUC | 0.5489428 | [0.4876335, 0.6061974] |
| Urine metabolomics | Accuracy | 0.7285897 | [0.6885897, 0.7600000] |
|  | BER | 0.4735632 | [0.4366667, 0.5102299] |
|  | AUC | 0.6119080 | [0.5326667, 0.7016322] |
| Stool metabolomics | Accuracy | 0.6363636 | [0.5393939, 0.7198106] |
|  | BER | 0.5291667 | [0.5000000, 0.5625000] |
|  | AUC | 0.6009259 | [0.5500000, 0.6435185] |

Table E3. Sparse Partial Least Squares Discriminant Analysis (sPLS-DA) confusion matrix in discriminating asthma and non-asthma diagnosis at age six based on stool microbiome, urine metabolome, and stool metabolome data.

| Data | Truth | Predicted | Count |
| --- | --- | --- | --- |
| Stool Microbiome | No | No | 319 |
| Stool Microbiome | Yes | No | 71 |
| Stool Microbiome | No | Yes | 10 |
| Stool Microbiome | Yes | Yes | 2 |
| Urine Metabolites | No | No | 139 |
| Urine Metabolites | Yes | No | 44 |
| Urine Metabolites | No | Yes | 10 |
| Urine Metabolites | Yes | Yes | 6 |
| Stool Metabolites | No | No | 34 |
| Stool Metabolites | Yes | No | 16 |
| Stool Metabolites | No | Yes | 7 |
| Stool Metabolites | Yes | Yes | 1 |

Table E4. Sparse Partial Least Squares Discriminant Analysis (sPLS-DA) selected Stool microbiome feature loadings

|  | **Feature** | **sPLS-DA Component 1** | **sPLS-DA Component 2** | **Weighted score** |
| --- | --- | --- | --- | --- |
| 1 | Massiliensis1 | -0.513914898 | 0 | 0.130048337 |
| 2 | Unclassified Lactococcus1 | 0 | 0.680476052 | 0.105031074 |
| 3 | Unclassified [Clostridium] innocuum group1 | -0.408558631 | 0 | 0.103387488 |
| 4 | Ramosum | 0 | 0.6667136 | 0.102906847 |
| 5 | Unclassified Lachnoclostridium1 | -0.395395714 | 0 | 0.100056556 |
| 6 | Unclassified Staphylococcus1 | 0.26113823 | 0 | 0.066082133 |
| 7 | Aerofaciens | -0.25502544 | 0 | 0.064535265 |
| 8 | Unclassified Dorea1 | -0.222545504 | 0 | 0.05631608 |
| 9 | Unclassified Streptococcus2 | -0.219084726 | 0 | 0.055440316 |
| 10 | Unclassified Veillonella1 | -0.176526016 | 0 | 0.044670654 |
| 11 | Difficile | -0.168536129 | 0 | 0.04264878 |
| 12 | Unclassified Bacteroides3 | -0.165585526 | 0 | 0.041902117 |
| 13 | Caccae2 | -0.152361961 | 0 | 0.038555838 |
| 14 | Unclassified Streptococcus6 | 0 | 0.233397503 | 0.036024766 |
| 15 | Merdae | -0.140065252 | 0 | 0.035444104 |

Table E5. Fifteen gut microbiome ASV sets relevant to asthma using Sparse Partial Least Squares Discriminant Analysis (sPLS-DA) were enriched in related disease/conditions.

| **Disease Term** | **Count** | **Percent** | **Fold** | **P-value** | **Bonferroni** | **FDR** |
| --- | --- | --- | --- | --- | --- | --- |
| Asthma | 1 | 0.33333333 | 8.88888889 | 0.10877123 | 1 | 0.2055 |
| Cystic fibrosis | 1 | 0.33333333 | 8.88888889 | 0.10877123 | 1 | 0.2055 |
| Diabetes mellitus type 1 | 5 | 0.03267974 | 0.87145969 | 0.81071662 | 1 | 0.8107 |
| Diabetes mellitus type 2 | 1 | 0.25 | 6.66666667 | 0.14261536 | 1 | 0.2424 |
| Interocolitis necrotizing | 2 | 0.22222222 | 5.92592593 | 0.03932943 | 0.6686 | 0.1639 |
| Irritable bowel syndrome | 3 | 0.2 | 5.33333333 | 0.01331089 | 0.2263 | 0.1131 |
| Liver cirrhosis | 5 | 0.09803922 | 2.61437908 | 0.02287671 | 0.3889 | 0.1296 |
| Periodontal diseases | 1 | 0.14285714 | 3.80952381 | 1 | 0.23740002 | 0.3363 |
| Psoriasis | 2 | 0.5 | 13.33333333 | 0.00723884 | 0.1231 | 0.1131 |
| Chronic obstructive | 1 | 0.2 | 5.33333333 | 0.17531223 | 1 | 0.2709 |
| Vaginosis bacterial | 1 | 0.05 | 1.33333333 | 0.54935932 | 1 | 0.5837 |

Table E6. Sparse Partial Least Squares Discriminant Analysis (sPLS-DA) selected urine metabolome feature loadings

| **Feature** | **sPLS-DA Component 1** | **sPLS-DA Component 1** | **Weighted score** |
| --- | --- | --- | --- |
| Argininate | 0.006063813 | 0.711454431 | 0.274607756 |
| Butenoylglycine | 0.046642571 | 0.61870797 | 0.23893277 |
| 3-hydroxyindolin-2-one | 0.727121014 | 0 | 0.120452516 |
| 2-methylhippurate | 0.679536462 | 0 | 0.112569813 |
| Kynurenine | 0 | -0.254846442 | 0.098365177 |
| 3-methyl-2-oxobutyrate | 0 | -0.155827571 | 0.06014605 |
| Glucuronide of C14H26O4 (1) | 0 | 0.147662111 | 0.05699436 |
| Benzoate | -0.085515659 | 0 | 0.014166248 |

Table E7. Sparse Partial Least Squares Discriminant Analysis (sPLS-DA) selected stool metabolome feature loadings

| **Feature** | **sPLS-DA Component 1** | **sPLS-DA Component 1** | **Weighted score** |
| --- | --- | --- | --- |
| Cholesterol | -0.855485854 | 0 | 0.249626658 |
| Fructose | 0.102514744 | 0.645726761 | 0.156675473 |
| PAHSA (16:0/OH-18:0) | 0.398850625 | 0 | 0.116382694 |
| Myristate (14:0) | 0 | -0.445395412 | 0.106080252 |
| 3,7-dimethylurate | 0 | 0.438782077 | 0.104505147 |
| 5,6-dihydrothymine | 0 | 0.362735915 | 0.08639316 |
| LAHSA (18:2/OH-18:0) | 0.269373195 | 0 | 0.078601803 |
| Palmitate (16:0) | 0 | -0.24607483 | 0.058607878 |
| Desmosterol | -0.161217066 | 0 | 0.047042365 |

Table E8. MetOrigin putative source assignment (prediction) for urine and stool metabolites that were significantly correlated with the microbiome taxa.

| **HMDBID** | **KEGGID** | **Name** | **Diff** | **Origin assignment** | **Source Links** |
| --- | --- | --- | --- | --- | --- |
| HMDB0000517 | C00062 | L-Arginine | 1 | Host; Microbiota; Food related; Drug related; Environment | Microbiota(BIGG:arg__L ChEBI:16467 KEGG:C00062,D02982); Food related(FooDB:FDB002257 HMDB:HMDB0000517 T3DB:T3D4289); Drug related(DrugBank:DB00125 KEGG:C00062,D02982 T3DB:T3D4289); Environment(HMDB:HMDB0000517 T3DB:T3D4289) |
| HMDB0000714 | C01586 | 2-Methylhippuricacid | 1 | Microbiota; Food related; Drug related; Environment | Microbiota(BIGG:bgly HMDB:HMDB0000714); Food related(FooDB:FDB001819 HMDB:HMDB0000714 T3DB:T3D4159); Drug related(DrugBank:DB16842); Environment(T3DB:T3D4159) |
| HMDB0001982 | C16360 | 3,7-Dimethyluricacid | 1 | Host; Microbiota; Food related | Microbiota(KEGG:C16360); Food related(FooDB:FDB022780) |
| HMDB0000079 | C00906 | 5,6-dihydrothymine | 1 | Microbiota; Food related | Microbiota(BIGG:56dthm); Food related(FooDB:FDB030555 HMDB:HMDB0000079 T3DB:T3D4332) |
| HMDB0000827 | C01530 | Stearicacid | 1 | Host; Microbiota; Food related; Drug related; Environment | Microbiota(BIGG:ocdca ChEBI:25629,28842 KEGG:C01530,D00119); Food related(FooDB:FDB031183 HMDB:HMDB0000827 T3DB:T3D4755); Drug related(DrugBank:DB03193 KEGG:C01530,D00119); Environment(HMDB:HMDB0000827 T3DB:T3D4755) |
| HMDB0000067 | C00187 | Cholesterol | 1 | Host; Microbiota; Food related; Drug related; Environment | Microbiota(BIGG:chsterol KEGG:C00187,D00040); Food related(FooDB:FDB013269 HMDB:HMDB0000067 T3DB:T3D4314); Drug related(DrugBank:DB04540 KEGG:C00187,D00040); Environment(HMDB:HMDB0000067 T3DB:T3D4314) |
| HMDB0002719 | C01802 | Desmosterol | 1 | Host; Microbiota; Food related | Microbiota(KEGG:C01802); Food related(FooDB:FDB005374 HMDB:HMDB0002719) |
| HMDB0112106 | NA | FAHFA(16:0/9-O-18:0) | 1 | Food related | Food related(HMDB:HMDB0112106) |
| HMDB0001348 | C00550 | SM(d18:1/18:0) | 1 | Host; Microbiota; Food related | Microbiota(KEGG:C00550); Food related(FooDB:FDB022570 HMDB:HMDB0001348) |
| HMDB0001348 | C00550 | SM(d18:0/14:0) | 1 | Host; Microbiota; Food related | Microbiota(KEGG:C00550); Food related(FooDB:FDB022570 HMDB:HMDB0001348) |
| HMDB0000950 | NA | Cer(d18:1/23:0) | 1 | Food related | Food related(FooDB:FDB022334 HMDB:HMDB0000950) |
